# Integrated One-Pot Enrichment and Immobilization of Styrene Monooxygenase (StyA) Using SEPABEAD EC-EA and EC-Q1A Anion-Exchange Carriers

**DOI:** 10.3390/molecules16075975

**Published:** 2011-07-18

**Authors:** Reto Ruinatscha, Rohan Karande, Katja Buehler, Andreas Schmid

**Affiliations:** Department of Chemical and Biochemical Engineering, Chair of Chemical Biotechnology, TU Dortmund, Dortmund 44221, Germany

**Keywords:** enzyme purification, enzyme immobilization, styrene monooxygenase, Sepabeads

## Abstract

A straightforward one-pot procedure combining enrichment and immobilization of recombinantely expressed FADH_2_ dependent styrene monooxygenase (StyA) directly from *Escherichia coli* cell extracts was investigated. Sepabeads EC-EA and EC-Q1A anion-exchange carriers were employed to non-covalently adsorb StyA from the cell extracts depending on basic parameters such as varying initial protein concentrations and pH. The protein fraction of the cell extract contained around 25% StyA. At low initial protein concentrations (2.5 mg mL^−1^) and pH 6, the enzyme could be enriched up to 52.4% on Sepabeads EC-EA and up to 46.0% on Sepabeads EC-Q1A, accounting for an almost complete StyA adsorption from the cell extracts. Higher initial protein concentrations were necessary to exploit the high loading capacity of the beads. At 20 mg mL^−1^, up to 37.6% of the theoretical bead loading capacity could be utilized for StyA binding using Sepabeads EC-EA, and 34.0% using Sepabeads EC-Q1A. For both carriers, protein leakage under reaction conditions could be reduced to less than 2%. During assays, the FADH_2_ cofactor necessary for StyA activity was supplied by the NADH-FAD reductase component styrene monooxygenase B (StyB). StyA immobilized on Sepabeads EC-Q1A displayed twice as high styrene epoxidation rates (0.2 U mg_StyA_^−1^) as compared to Sepabeads EC-EA. This activity could be increased to 0.7 U mg_StyA_^−1^ by co-immobilizing StyB on Sepabeads EC-Q1A, which corresponds to 33% of the soluble StyA activity.

## 1. Introduction

The capability of enzymes to catalyze reactions at high efficiencies, extraordinary selectivities, and under ambient, energy-saving conditions has made them potential alternatives over chemical catalysts in production processes [1,2,3,4,5]. Despite their usefulness for a given reaction, the economic application of isolated enzymes is often hampered by insufficient long-term stabilities [6], as well as high production and enrichment costs [7]. A general solution to improve enzyme economics is immobilization. Immobilized enzymes typically display enhanced operational stabilities and can easily be separated from reaction mixtures, allowing for their repetitive, cost-efficient use [8,9,10,11].

In biotransformations using immobilized enzymes, overall process costs per product quantity can be reduced considerably if enzymes are enriched prior to immobilization [12]. However, the enrichment of intracellular enzymes frequently requires additional purification matrixes and chromatographic instruments, and often consumes large quantities of buffers, salts, or solvents. Such multistage procedures not only affect process costs adversely, but also result in lowered enzyme yields and extended preparation times. This is especially critical for labile enzymes that are prone to inactivation prior to immobilization.

Therefore, fast and inexpensive procedures combining enrichment and immobilization from cell extracts in one operational step would be of high interest for industrially relevant enzymes. Affinity fusion strategies, which are well known to selectively adsorb proteins from cell extracts [13,14,15,16], have already successfully been applied to combine enzyme enrichment with immobilization for subsequent biocatalysis [17]. Unfortunately, the introduction of affinity tags is not applicable to every enzyme. Affinity tags sometimes interfere with protein folding, leading to inactive or poorly soluble enzymes, or may be buried within the protein structure, which renders them inaccessible for the corresponding affinity ligands on the immobilization carriers [18,19]. In addition, the costs of affinity carriers are often too high to be applied in industrial biotransformations. A solution to overcome expensive carriers has recently been suggested for lipases. Due to their comparably high surface hydrophobicity, these enzymes can selectively be enriched from cell extracts on conventional hydrophobic supports in one operational step [20,21]. In turn, this technique is only applicable for enzymes having similar surface properties as lipases.

The focus of this work was therefore to investigate a more general and less expensive procedure aimed at combining enzyme enrichment and immobilization directly from the cell extract in one operational step. Cheap and commercially available Sepabead EC-EA and EC-Q1A anion-exchange carriers were employed for the non-covalent enzyme immobilization via adsorption. Due to the reversible enzyme binding, these supports can be recovered after repetitive catalytic cycles, and reused for follow-up immobilizations [22]. Immobilizations were carried out in batch mode and dependent on basic parameters such as pH and initial cell extract protein concentration. The monooxygenase component StyA from the two-component styrene monooxygenase enzyme system StyAB from *Pseudomonas sp.* strain VLB120 [23], recombinantely synthesized in *Escherichia coli* JM101, was used as a model enzyme. StyA (*S*)-epoxidizes the vinyl side chain of various unfunctionalized olefins at enantiomeric excesses higher than 98% [24], which makes it interesting for industrial applications. StyA catalyzed epoxidations are dependent on molecular oxygen and the flavin cofactor FADH_2_ as redox equivalent [23]. Since FADH_2_ rapidly re-oxidizes under aerobic conditions [25] it needs to be continuously regenerated in order to sustain the epoxidation reaction. FADH_2_ regeneration was achieved using the NADH-FAD reductase component styrene monooxygenase B (StyB). StyB was either directly added to reaction media containing immobilized StyA, or co-immobilized on the carriers containing StyA, in order to elucidate possible StyAB-interactions necessary for efficient FADH_2_ transfer during epoxidation catalysis.

## 2. Results and Discussion

StyA was directly immobilized from the clarified cell extracts on commercially available Sepabeads via adsorption. The spherical, highly porous carriers were employed with ethylamino functionalizations (Sepabeads EC-EA), as well as with quaternary ammonium functional groups (Sepabeads EC-Q1A), which both are positively charged under physiological pH conditions. The immobilization conditions were chosen based on the pH dependent ionization state of StyA. 2D SDS-PAGE gave an isoelectric point (pI) of 5.2, which was in good agreement with the theoretically calculated value (5.3 ± 0.4) [26]. In order to promote adsorption of negatively ionized StyA on the carriers, and since protein solubilities are lowest at their isoelectric point [27], the cell extracts were subject to immobilization at pH 6, 6.5, and 7.

Pefabloc was added prior to the immobilization procedures in order to minimize the degradation of cell extract proteins by cellular proteases. The time course of the adsorptions was not influenced by the protease inhibitor. For both Sepabead carriers, adsorption equilibria between bound and unbound proteins in the supernatant were always reached within 3 h, independent of the conditions applied. After the immobilization supernatants were clear and did not indicate any protein precipitation. Based on these observations we concluded that the decreasing protein supernatant concentrations and changes in the soluble StyA fraction in presence of the beads were the result of adsorption rather than side reactions. This was supported by control experiments without beads, showing that volumetric StyA activities did not change more than 5% during 3 h exposed to immobilization conditions (data not shown).

### 2.1. One-Pot Immobilization of Recombinant StyA from *Escherichia coli* JM101 (pSPZ10) Cell Extracts on SEPABEADS® EA and Q1A

**Figure 1:**
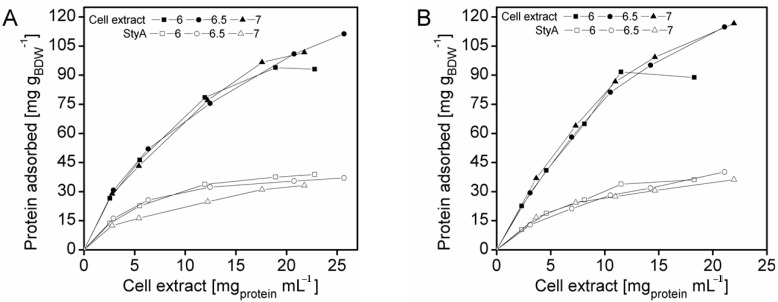
StyA enrichment from *Escherichia coli* JM101 (pSPZ10) cell extract at various pH values on (**A**) Sepabeads EC-Q1A and (**B**) Sepabeads EC-EA. StyA enrichments reflect the fraction of adsorbed StyA over totally adsorbed *Escherichia coli* JM101 cell extract proteins at binding equilibrium.

To enrich StyA on the carriers, adsorption studies were investigated at protein concentrations up to 27.3 mg mL^−1^ and at pH 6, 6.5 and 7. The total amount of protein adsorbed on the beads was calculated by the difference between the initial and final amount of enzyme in the supernatant, while the amount of StyA adsorbed to the beads was determined by quantifying StyA activity in the crude extract before and after immobilization. At pH 6 the beads reached maximum protein binding capacities at crude extract concentrations of 18 mg mL^−1^ and 12 mg mL^−1^ for Q1A and EA beads respectively (Figure 1). In contrast, for pH 6.5 and pH 7, adsorption on both beads still took place, even at crude extract concentrations above 20 mg mL^−1^. The adsorption rate of StyA from the crude extract on Q1A was lower at pH 7 compared to pH 6.5 and 6. Whereas, the adsorption rate of StyA on EA was similar, regardless of pH. At higher crude extract concentrations adsorption of foreign proteins was still continuing while StyA adsorption leveled off. For the detailed analysis, the maximum protein adsorption capacities on the beads were estimated using the Langmuir adsorption isotherm.

### 2.2. Calculation of Maximal Bead Capacity According to the Langmuir Adsorption Isotherm

Isothermal batch adsorptions of proteins on anion-exchange carriers are often described by the Langmuir model (Equation 1), enabling the theoretical determination of maximal adsorption capacities with respect to the applied carriers:

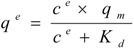
(1)
where c^e^ is the soluble protein concentration at binding equilibrium, q^e^ the protein concentration adsorbed on the carriers at binding equilibrium, K_d_ the dissociation constant for the protein-carrier complex, and q_m_ the maximal adsorption capacity of the carriers. Equation 1 was rearranged to generate the linear form:

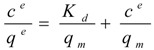
(2)

For solutions containing a single protein these capacities are reported to be in good agreement with experimentally determined values according to [28,29,30,31]. Interestingly, also the complex mixture of cell extract proteins (Figure 1) showed a concentration dependent adsorption behaviour that could be linearized according to Langmuir isotherm for both, the total cell extract proteins and StyA. The calculated maximal adsorption capacities were 6 to 13% higher for Sepabeads EC-EA than for Sepabeads EC-Q1A, depending on the pH (Table 1). This observation is possibly a result of the different functional group densities, which is 20% higher for Sepabeads EA compared to Sepabeads Q1A (Resindion S.R.L.). The maximal adsorption capacities with respect to the total cell extract proteins strongly increased with the pH values, while StyA specific maximal adsorption capacities were calculated to be in the range of 40 mg_StyA_ g_BDW_^−1^ at pH 6, and 44 mg_StyA_ g_BDW_^−1^ at pH 6.5 and 7 (Table 1). Apparently, the net charge of StyA only slightly affected its binding to the carriers, whereas cell extract proteins other than StyA could selectively be excluded from binding depending on pH. This is insofar not astonishing as the *Escherichia coli* proteome profiles reveals considerable pI heterogeneity ranging from 4 to 12 [32].

**Table 1:** Calculated maximum protein adsorption capacities of Sepabeads EC-EA and Sepabeads EC-Q1A according to Langmuir isotherms. Linearizations were performed for the total cell extract proteins and StyA at pH 6, 6.5, and 7. The regressed R^2^ values for each linear fit were consistently greater than 0.98.

	Sepabeads EC-EA		Sepabeads EC-Q1A	
pH	total cell extract proteins [mg g_BDW_^−1^]	StyA [mg g_BDW_^−1^]	total cell extract proteins [mg g_BDW_^−1^]	StyA [mg g_BDW_^−1^]
6	96	41	114	39
6.5	141	44	133	44
7	152	43	132	44

BDW: bead dry weight.

### 2.3. StyA Enrichment on the Carriers, Immobilization Efficiency, and Bead Capacity Usage

**Figure 2:**
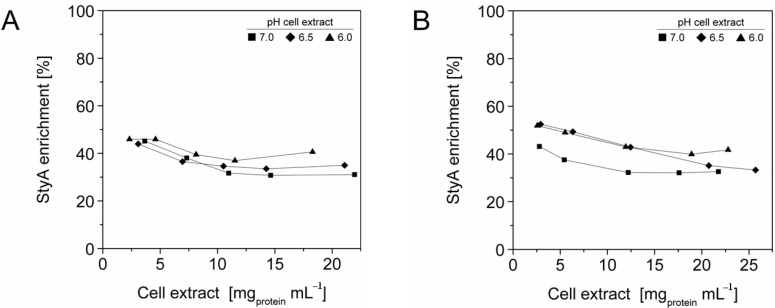
StyA enrichment from *Escherichia coli* JM101 (pSPZ10) cell extract at various pH values on (**A**) Sepabeads EC-EA and (**B**) Sepabeads EC-Q1A. StyA enrichments reflect the fraction of adsorbed StyA over totally adsorbed *Escherichia coli* JM101 cell extract proteins at binding equilibrium.

**Figure 3:**
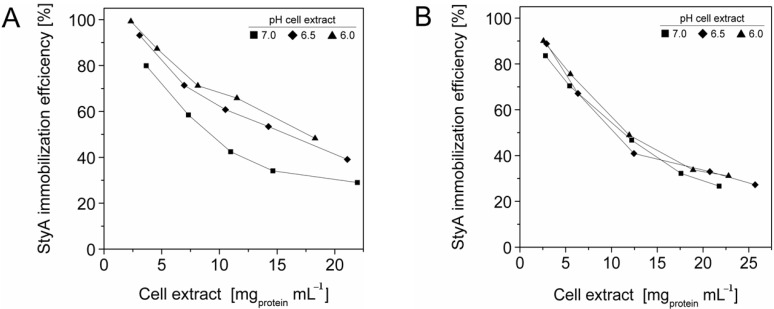
StyA immobilization efficiency from *Escherichia coli* JM101 (pSPZ10) cell extract on (**A**) Sepabeads EC-EA and (**B**) Sepabeads EC-Q1A at various pH values. Efficiencies are based on the ratios of adsorbed over soluble StyA at binding equilibrium.

**Figure 4:**
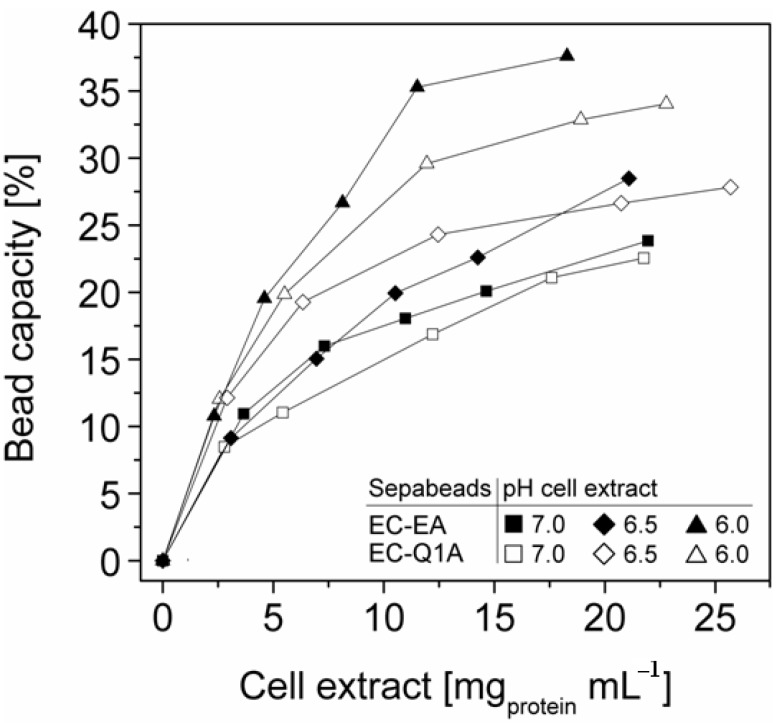
StyA bead capacity usage as a function of initial cell extract protein concentration and pH. The maximum adsorption capacities were calculated based on Langmuir isotherms (Table 1).

The efficient and selective usage of the carriers to enrich and immobilize StyA (Figure 1) was further analyzed by estimating StyA enrichment (Figure 2), immobilization efficiency (Figure 3) and bead capacity usage (Figure 4). At pH 7 and high initial protein concentrations, the fraction of StyA was on both Sepabead carriers around 30%, which is close to the relative StyA protein fraction in *Escherichia coli* JM101 (pSPZ10) cell extracts (25%) [33]. Highest StyA enrichments were determined at low initial protein concentrations (2.5 mg mL^−1^) at pH 6, being in the range of 52.4% and 46.0% for Sepabeads EC-EA and Q1A, respectively (Figure 2). Under these conditions, the immobilization efficiency with respect to the amount of StyA withdrawn from the cell extracts was higher than 90% (Figure 3). Overall, StyA enrichment on the carriers and adsorption efficiencies from the cell extracts was highest at low initial protein concentrations and pH values close to the pI of StyA. However, under these conditions only around 10% of the bead capacity could be exploited for StyA binding based on the calculated maximal adsorption capacities (Table 1).

More efficient utilizations of the carriers, which may be advantageous in biocatalytic applications requiring high catalyst densities, were achieved at elevated initial cell extract protein concentrations and low pH values. In case of Sepabeads EC-EA, up to 37.6% of the calculated binding capacity was utilized by StyA at pH 6 and at an initial protein concentration of 18.3 mg mL^−1^. Similarly, at pH 6 and at the highest protein concentration investigated with Sepabeads EC-Q1A (22.8 mg mL^−1^), up to 34.0% of the theoretical bead capacity could be occupied by StyA (Figure 4).

### 2.4. Immobilized StyA stability

**Figure 5:**
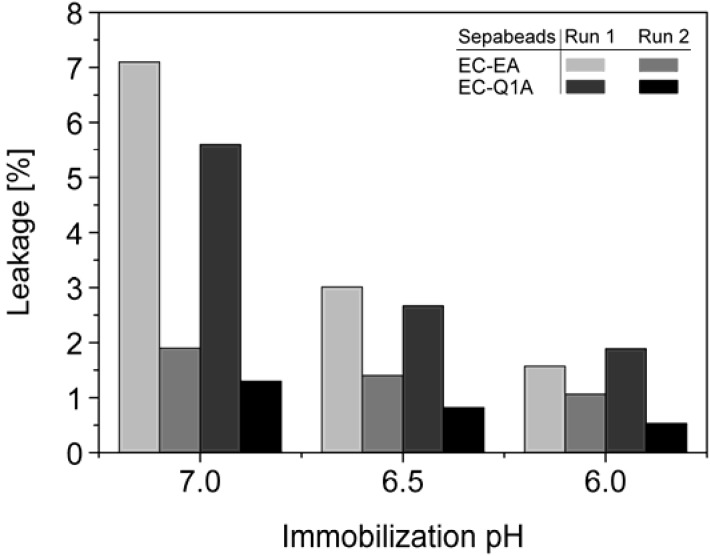
Total leakage of immobilized StyA and *Escherichia coli* JM101 (pSPZ10) cell extract proteins during 1 h under reaction conditions (pH 7.5) as a function of the pH during immobilization. Protein leakage was investigated in two successive steps with the same beads.

StyA stability of different immobilization batches (pH 6, 6.5, and 7) was investigated with respect to protein leakage from the carriers under reaction conditions (pH 7.5). The concentration of desorbed proteins was determined after two successive reaction cycles. Proteins adsorbed on Sepabeads EC-EA were more prone to leakage than the ones on Sepabeads EC-Q1A, despite their higher functional group density and the stronger Coulomb interactions possible. Chemical functionalities of the spacer arms were obviously also involved in protein retention. Similar observations are reported for cation exchange adsorbents, where spacer arm variations have been shown to significantly influence protein retention [34]. Total protein leakage was highest during the first reaction cycle. For the proteins immobilized at pH 7 up to 7% desorbed, while only 2% of the proteins immobilized at pH 6 came off the carriers under the same reaction conditions. Apparently, increasing pH differences between immobilization and reaction conditions were beneficial for intensified protein-carrier interactions. After the second leakage run less than 2% of immobilized proteins came off the carriers, independent of the pH during immobilization (Figure 5). Overall, the results indicate that StyA on either of the two Sepabeads may be reused for several catalytic cycles without significant loss of enzyme, especially if immobilizations are carried out at pH 6.

### 2.5. Biocatalytic Activity of Immobilized StyA

In order to minimize background activity of desorbed, soluble StyA, immobilizations were carried out at pH 6. Immobilized StyA activities were determined using the NADH-FAD reductase component StyB, delivering the FADH_2_ cofactor necessary for StyA activity [23]. Initially, StyB was added to the reaction medium containing immobilized StyA and the time-course of styrene epoxidation was directly analyzed. Under these conditions specific styrene epoxidation activities of StyA immobilized on Sepabeads EC-Q1A were 0.2 U mg_StyA_^−1^, and 0.1 U mg_StyA_^−1^ for StyA immobilized on Sepabeads EC-EA. The observed activities possibly correlate with the chemical composition of the Sepabead carriers. Whereas Sepabeads EC-EA consists of a polymethacrylate matrix, Sepabeads EC-Q1A are composed of a polystyrene framework, which could provide a catalytically advantageous microenvironment for the styrene dependent styrene monooxygenase. It may also be speculated that less strong interactions of the Sepabeads EC-Q1A quaternary amino groups with StyA enabled a higher degree of structural freedom beneficial for retaining an active enzyme conformation during catalysis [35], unlike with the ethylamino functionalized Sepabeads EC-EA.

However, the observed activities were rather low compared to the soluble StyA activity, which is reported to be 2.1 U mg_StyA_^−1^ [23]. Over the past years it has been speculated that reductive and oxidative flavin components, such as StyB and StyA, form a transient complex during catalysis, thereby protecting reduced flavin from counterproductive uncoupling reactions with free oxygen and oxidized flavin in solution [36,37]. Accordingly maximal StyA activities in solution increase until the molar StyB:StyA ratios are equal [23]. It is very likely that the spatial orientation between immobilized StyA and soluble StyB was impaired, and that FADH_2_ transfer between the two enzyme components rather occurred via diffusion than complex-mediation. It thus appears that the observed low StyA activities were the result of FADH_2_ limitation.

**Figure 6:**
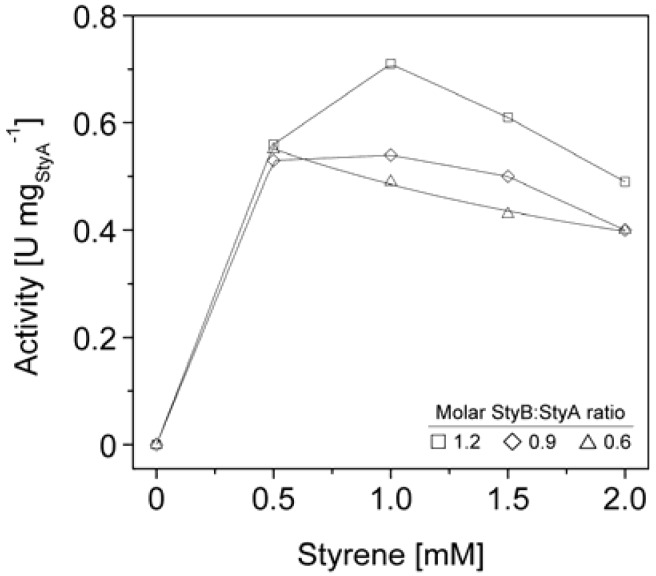
StyA activities immobilized from *Escherichia coli* JM101 (pSPZ10) cell extract on Sepabeads EC-Q1A as a function of molar StyB:StyA ratios and styrene concentrations.

We therefore co-immobilized StyB on the Sepabeads EC-Q1A carriers containing StyA to establish close proximity of the two enzyme components. Similar to the soluble StyAB enzyme system, styrene epoxidation rates increased with the molar StyB:StyA ratios immobilized on the carriers. At a molar ratio of 1.2 up to 0.7 U mg_StyA_^−1^ were achieved (Figure 6), corresponding to 33% of the soluble StyA epoxidation activity [23]. This activity compares well with immobilized enzymes performing oxidation reactions, which typically retain up to 6% of their soluble activity [38,39,40]. Apart from the molar StyB:StyA ratios, activities of the immobilized enzyme system were also dependent on the initial styrene concentrations. Maximal activities were measured in the range of 0.5 to 1 mM styrene. Interestingly, the decrease in epoxidation activity got more significant with increasing molar StyB:StyA ratios.

The styrene epoxidation activity of 0.2 U mg_StyA_^−1^ with soluble StyB and immobilized StyA on Sepabeads EC-Q1A was achieved at a molar StyB:StyA ratio of 1 and a styrene concentration of 2 mM. Under these conditions, styrene epoxidation rates with co-immobilized StyB were at least two fold higher (Figure 6). We assume that the close proximity of StyA and StyB on the carrier enabled shorter FADH_2_ diffusion distances compared to the reaction system with StyB in solution. As a result, FADH_2_ uncoupling was less probable, leading to higher residual FADH_2_ concentrations available for StyA epoxidation.

## 3. Experimental

### 3.1. Materials

Sepabeads EC-EA (batch SC 5O 6/387) and Sepabeads EC-Q1A (batch SR 7Z 5C101) were kindly donated by Resindion S.R.L. (Binasco, Italy). StyB synthesis and purification was performed as described in literature [23]. All chemicals and enzymes obtained commercially were of the highest grade available unless otherwise indicated. Acetonitrile was purchased from Fischer Scientific GmbH (Schwerte, Germany). Trypsin was acquired from Promega (Mannheim, Germany). 2D SDS-PAGE was performed according to the method of O'Farrell [41]. All necessary components were bought from Invitrogen GmbH (Karlsruhe, Germany). Protein quantification according to Bradford [42] was carried out using the Quick Start kit from Bio-Rad Laboratories GmbH with bovine serum albumin (Sigma-Aldrich, Steinheim, Germany) as standard. The remaining reagents were purchased from Sigma-Aldrich (Steinheim, Germany). Buffers and solutions were prepared with de-ionized water according to Beynon and Easterby [43].

### 3.2. One-Pot Immobilization of Recombinant StyA from *Escherichia coli* JM101 (pSPZ10) Cell Extracts

The StyA gene was overexpressed in *Escherichia coli* JM101 (pSPZ10) as described in literature [23]. Cells (1 g wet weight) were dissolved in 50 mM Bis-Tris (5 mL, pH 6.0, 6.5 or 7.0 at 15 °C) containing 1 mM DTT and 1 mM Pefabloc. The solubilized cells were disrupted by two passages through a French press unit (Aminco SLM Instruments, Urbana, IL, USA) at 1,050 psi. Insoluble cell debris was removed by ultracentrifugation in a Sorvall Discovery 90SE centrifuge (Kendro Laboratory Products GmbH, Langenselbold, Germany) for 45 min at 91,500 × g (4 °C). The obtained clarified cell extracts were adjusted to protein concentrations ranging from 3 mg mL^−1^ to 25 mg mL^−1^ with the same buffer used to dissolve the cells. For immobilization, portions (1,600 µL) were added to Sepabeads EC-EA or Sepabeads EC-Q1A (100 mg dry weight—BDW) in 2 mL Eppendorf safe-lock tubes (Eppendorf, Hamburg, Germany). Immobilizations were carried out in a Heidolph REAX 2 overhead shaker (Heidolph Instruments GmbH & Co. KG, Schwabach, Germany) at 23 rpm (15 °C). The procedures were stopped after 3 h (adsorption equilibrium). The amount of immobilized proteins was calculated from the mass-balance between the initial and the residual protein concentration in the supernatant.

### 3.3. Determination of StyA Enrichment on the Carriers and Immobilization Efficiency

StyA enrichment on both Sepabead carriers (relative StyA protein amount on the carriers) and immobilization efficiency (relative StyA protein amount adsorbed from the cell extracts) was quantified by comparing styrene epoxidation activities before immobilization with the same volume of cell extract solution after immobilization. The activity assays were adjusted to a final protein concentration of 100 µg mL^−1^ in 1 mL of reaction mixture. Activity assays were carried out at 37 °C in 50 mM Tris buffer (pH 7.5) supplemented with 1 mM DTT (2 mL Eppendorf safe-lock tubes). A typical reaction mixture consisted of 150 mM sodium formate, 15 µM FAD, 0.5 U formate dehydrogenase, 650 U catalase, and 37 µg StyB, corresponding to a molar ratio of 1 with respect to the initial cell extract protein (before immobilization). The mixtures were pre-incubated in a thermo mixer for 2.5 min (300 rpm, 37 °C). Subsequently, NADH and styrene were added to a final concentration of 5 mM and 2 mM, respectively. The reactions were carried out at 37 °C in a vertically positioned thermo mixer (1,400 rpm). After 0.5 to 3 min, the reactions were stopped by the addition of 1 mL ice cold acetonitrile and mixed in a vertically positioned thermo mixer for 2 min (1,400 rpm, 10 °C). The obtained mixtures were centrifuged for 5 min (4 °C, 16200 × g) in a Heraeus Fresco 17 Microcentrifuge (Thermo Electron Corporation, Langenselbold, Germany) and analyzed by reversed phase HPLC. Up to 5 independent activity assays were carried out for each StyA concentration determination.

### 3.4. Leakage of Immobilized Proteins

StyA was directly immobilized from *Escherichia coli* JM101 (pSPZ10) cell extract containing 4.75 mg mL^−1^ protein, as described in Section 2.2. Subsequently, the beads were washed five times with fresh immobilization buffer. Protein leakage was investigated at the same bead concentration as during immobilization, using 50 mM Tris buffer pH 7.5 (1 mM DTT, 37 °C). After 1 h of vertical agitation at 1,400 rpm in an Eppendorf thermo mixer comfort (Eppendorf, Hamburg, Germany) the desorbed protein was quantified by determining the protein concentrations in the supernatant. This procedure was repeated once.

### 3.5. Activity of Immobilized StyA in the Presence of Soluble StyB

StyA was directly immobilized on Sepabeads EC-EA and Sepabeads EC-Q1A from *Escherichia coli* JM101 (pSPZ10) cell extract containing 4.75 mg mL^−1^ protein in 50 mM Bis-Tris (pH 6.0), as described in Section 2.2. Subsequently, the beads were washed five times with fresh immobilization buffer. Activity assays were carried out as described in Section 2.3, using 20 mg of beads (wet weight) in the presence of soluble StyB (molar StyB:StyA ratio of 1).

### 3.6. Activity of Immobilized StyA in the Presence of co-Immobilized StyB

StyA was directly immobilized on Sepabeads EC-Q1A from *Escherichia coli* JM101 (pSPZ10) cell extract containing 4.75 mg mL^−1^ protein in 50 mM Bis-Tris (pH 6.0), as described in Section 2.2. Subsequently, the beads were washed five times with fresh immobilization buffer. StyB was co-immobilized to probe for StyA activity by adding 0.3 mL StyB (4 mg mL^−1^ in immobilization buffer) to 100 mg_BDW_ in 2 mL Eppendorf safe-lock tubes. The tubes were incubated at 15 °C (700 rpm) in a horizontally positioned thermo mixer. Varying molar StyB:StyA co-immobilization ratios were adjusted by changing immobilization times (ratio 0.6:20 min; ratio 0.9:30 min; ratio 1.2:55 min). After co-immobilization, the supernatants were removed and the beads were washed with 50 mM Tris buffer (1 mM DTT, pH 7.5 at 37 °C). Activity assays were carried out as described in Section 2.3, using 20 mg of beads (wet weight) containing StyA and StyB. Styrene was added to a final concentration of 0.5 mM to 2 mM.

### 3.7. Analytical Procedures

Concentrations of styrene and styrene oxide were determined by HPLC on a LaChrom Elite Merck-Hitachi system (Darmstadt, Germany) equipped with a diode array detector and reverse phase CC 25014 Nucleosil 100-5 C18 HD (Machery-Nagel, Oensingen, Switzerland) column. Injected sample volumes were 20 µL, the mobile phase consisted of water and acetonitrile (ratio 60:40), and elution was isocratic (1 mL min^−1^) and isothermal (25 °C). The substances were identified by comparing the retention times to commercially available standards and quantified by means of standard curves, which were recorded under identical conditions.

The isoelectric point (pI) of StyA was determined by 2D SDS-PAGE. 10 µL of StyA (4.75 mg mL^−1^) were added to 155 µL sample rehydration buffer and absorbed overnight onto 7 cm pH 3–10 nonlinear immobilized pH gradient (IPG) ZOOM strips. Isoelectric focusing was performed using the ZOOM IPGRunner system and a Bio-Rad 3000 V power supply (Bio-Rad Laboratories GmbH, Munich, Germany). The following voltage steps were applied: 100 V for 30 min, 200 V for 20 min, 450 V for 15 min, 750 V for 15 min, and 2000 V for 30 min. For the second dimension, focused IPG strips were equilibrated in NuPAGE LDS sample buffer supplemented with NuPAGE sample reducing agent for 15 min. Afterwards the stripes were incubated in LDS sample buffer containing 125 mM iodoacetamide for 15 min, and loaded onto 4–12% Bis-Tris gels embedded in 0.5% agarose (w/v). SDS-PAGE was performed at 120 V for 90 min.

## 4. Conclusions

Introducing simple, fast and efficient operation units at early stage production levels may positively impact overall process economics. We addressed this challenge for the straightforward enrichment and immobilization of recombinant StyA from *Escherichia coli* JM101 cell extracts. A two-fold enrichment of StyA in a simple one-step batch system was accomplished, while at the same time immobilizing the enzyme at nearly 100% efficiency from the cell extracts, and making use of up to 37.6% of the theoretical bead capacity, depending on the conditions applied. One of the decisive parameters was the pH, which was preferably close to the isoelectric point of StyA. Comparably low pH values during immobilization could also significantly reduce protein leakage under reaction conditions to less than 2%. A simple way to tightly attach the proteins to the carriers would be post-treatment with glutaraldehyde. StyA immobilized on Sepabeads EC-Q1A retained 33% of its specific activity at a 1.2 fold molar excess of co-immobilized StyB. Since the dependency of StyB on styrene epoxidation rates was not fully elucidated, this activity could even be higher. Overall, we showed that enzyme enrichment and immobilization for biotransformations can be combined in one operational step. The here presented procedure is fast, inexpensive, requires no specialized reagents or equipments, and can easily be up-scaled. Thus, it can be anticipated that this procedure is well applicable to a variety of enzymes.
